# Mutations conferring SO_4_^2−^ pumping ability on the cyanobacterial anion pump rhodopsin and the resultant unique features of the mutant

**DOI:** 10.1038/s41598-022-20784-6

**Published:** 2022-09-30

**Authors:** Yuhei Doi, Jo Watanabe, Ryota Nii, Takashi Tsukamoto, Makoto Demura, Yuki Sudo, Takashi Kikukawa

**Affiliations:** 1 School of Science, Hokkaido University, Sapporo, 060-0810 Japan; 2 Graduate School of Life Science, Hokkaido University, Sapporo, 060-0810 Japan; 3 Faculty of Advanced Life Science, Hokkaido University, Sapporo, 060-0810 Japan; 4 Graduate School of Medicine, Dentistry and Pharmaceutical Sciences, Okayama University, Okayama, 700-8530 Japan

**Keywords:** Bioenergetics, Ion transport, Membrane proteins

## Abstract

Membrane transport proteins can be divided into two types: those that bind substrates in a resting state and those that do not. In this study, we demonstrate that these types can be converted by mutations through a study of two cyanobacterial anion-pumping rhodopsins, *Mastigocladopsis repens* halorhodopsin (MrHR) and *Synechocystis* halorhodopsin (SyHR). Anion pump rhodopsins, including MrHR and SyHR, initially bind substrate anions to the protein center and transport them upon illumination. MrHR transports only smaller halide ions, Cl^-^ and Br^-^, but SyHR also transports SO_4_^2−^, despite the close sequence similarity to MrHR. We sought a determinant that could confer SO_4_^2−^ pumping ability on MrHR and found that the removal of a negative charge at the anion entrance is a prerequisite for SO_4_^2−^ transport by MrHR. Consistently, the reverse mutation in SyHR significantly weakened SO_4_^2−^ pump activity. Notably, the MrHR and SyHR mutants did not show SO_4_^2−^ induced absorption spectral shifts or changes in the photoreactions, suggesting no bindings of SO_4_^2−^ in their initial states or the bindings to the sites far from the protein centers. In other words, unlike wild-type SyHR, these mutants take up SO_4_^2−^ into their centers after illumination and release it before the ends of the photoreactions.

## Introduction

In biological membranes, various membrane transporters fulfill vital roles by transporting specific substrates. For selective transport, these proteins have “tailor-made” machineries to allow their respective substrates to bind and pass through. For most transporters, these machineries have somewhat loose substrate selectivity and thus allow the transport of compounds with similar structures. In contrast, inorganic ion transporters require highly tuned binding sites and/or filters due to the tiny sizes of the substrates. Thus, it is an interesting challenge to identify the protein moieties responsible for ion selection and then modify the substrate ion specificities. We herein report this kind of challenge for anion transporting microbial rhodopsins.

Rhodopsins are photoactive membrane proteins that are widespread in various organisms^1^. They commonly consist of seven transmembrane helices and the chromophore retinal bound to a specific Lys residue via a Schiff base linkage. Illumination induces the isomerization of retinal, which in turn triggers sequential protein conformation changes. During this photochemical reaction, rhodopsins exhibit their respective functions. The rhodopsins of animals function mostly as light sensors. In contrast, microbial rhodopsins function not only as light sensors but also as ion pumps, ion channels, and even enzymes^1–4^. Concerning the pumps for chloride ions, they are divided into three main groups. The first and second Cl^−^ pumps were found in extremely halophilic archaea^5,6^ and marine eubacteria^7^, respectively. The archaeal Cl^−^ pumps are called halorhodopsin (HR), whereas the marine bacterial Cl^−^ pumps are sometimes referred to as Cl^−^ pumping rhodopsins (ClRs). These pumps strongly transport smaller halide ions, Cl^−^ and Br^−^, whereas with weaker efficiencies, they can also transport larger ions, I^−^ and NO_3_^−^. There are no precise reports on the residues responsible for anion selectivity in these Cl^−^ pumps. The third Cl^−^ pump was discovered in cyanobacteria, most of which inhabit terrestrial environments^8^. We herein examined this cyanobacterial Cl^−^ pump because of its advantages for this study, as described below.

**Figure 1:**
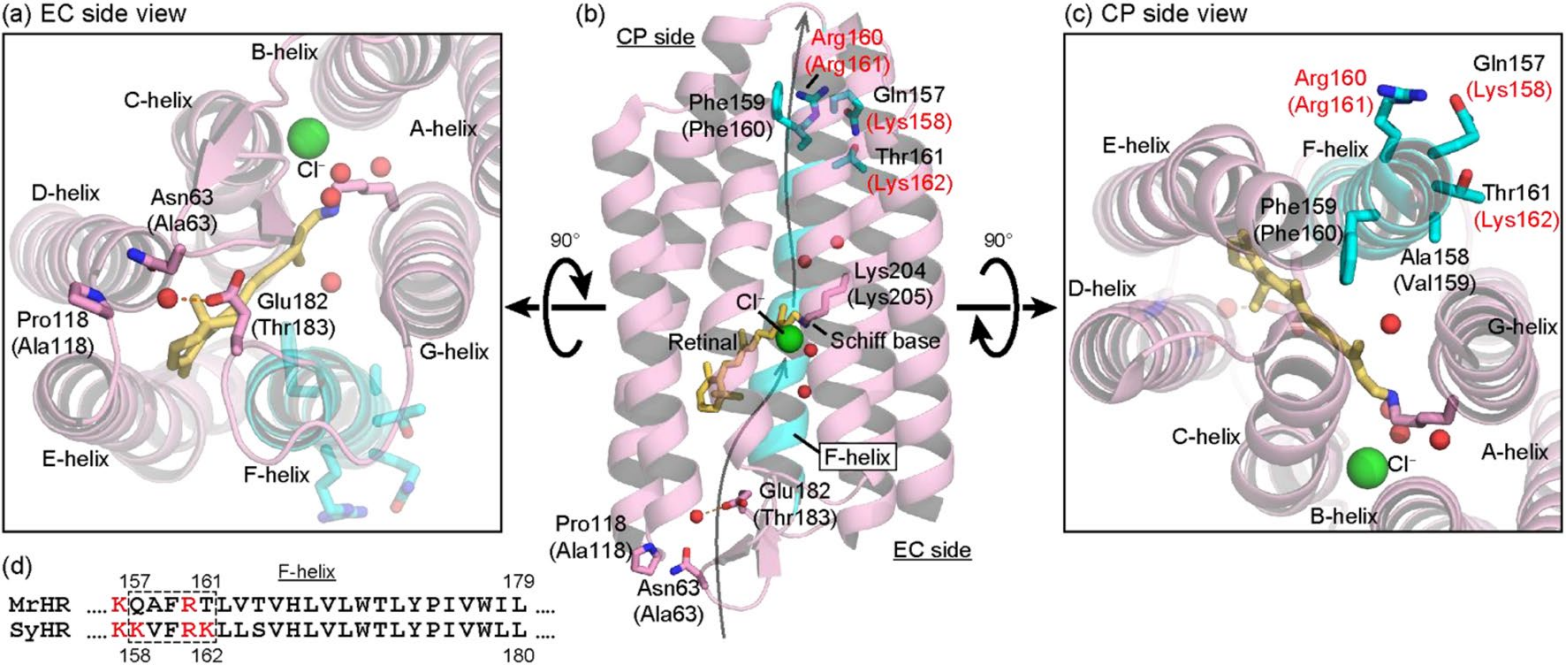
The positions of key residues in this study. The overall structure of MrHR is shown in (**b**) with the residues mutated in this study, and expanded views from the EC and CP sides are shown in (**a**) and (**c**), respectively (PDB code, 6K6I). The corresponding residues in SyHR are indicated in parentheses, and water molecules inside the protein are shown as red spheres. Out of seven helices, only the F-helix is colored light blue. In Panel (**b**), a putative anion transport pathway is indicated with gray arrows. (**d**) The amino acid residues in the F-helix are compared between MrHR and SyHR. In the CP end regions (the upstream region), SyHR has more basic residues (red letters) than MrHR. The five residues enclosed by the dashed box, which are shown with blue sticks in the upper panels, are mutually replaced between MrHR and SyHR. This mutation is referred to as “F-helix”.

For the cyanobacterial Cl^−^ pump, the first characterized member was a microbial rhodopsin from *Mastigocladopsis repens* and named *M. repens* halorhodopsin (MrHR)^8^. Later, another member named *Synechocystis* halorhodopsin (SyHR) was revealed to have a distinct difference from MrHR in terms of transportable ions^9^: MrHR pumps only smaller halide ions, Cl^−^ and Br^−^. However, SyHR can also pump SO_4_^2−^ despite high sequence similarity with MrHR (67% identity; 80% similarity). SO_4_^2−^ is a significantly larger ion than Cl^−^ and Br^−^, and SO_4_^2−^ transport was never observed for archaeal and marine eubacterial Cl^−^ pumps. The high sequence similarity between MrHR and SyHR is advantageous for identifying the site responsible for ion selectivity. Thus, we explored the mutation that confers SO_4_^2−^ pumping activity on MrHR and concluded that the negative charge at Glu182, which is located at the extracellular (EC) surface (Fig. 1a,b), is a key determinant of SO_4_^2−^ transport ability. Unexpectedly, the subsequent characterization of the mutant revealed unusual features. During this study, two research groups independently solved the tertiary structure of MrHR^10,11^. Out of them, Yun and coworkers also reported the mutations that confer SO_4_^2−^ pump activity^10^. Like us, they also mutated Glu182. However, they obtained the strongest SO_4_^2−^ activity by replacing residues in the vicinity of Glu182. These mutations did not include the removal or introduction of any charged residues. Thus, we continued mutation studies on MrHR and examined “reverse” mutations in SyHR to lower its SO_4_^2−^ pumping activity. We herein summarized our mutation results and the unique features of the MrHR and SyHR mutants. Wild-type MrHR, SyHR and all other anion-pumping rhodopsins bind the substrate anions to the vicinity of the Schiff base in the respective dark states. However, the MrHR and SyHR mutants seem to start the SO_4_^2−^ pumping reactions without the initial bindings of SO_4_^2−^ to their central parts.

## Results

### Reduction of light-induced artifact from the pH electrode

**Figure 2:**
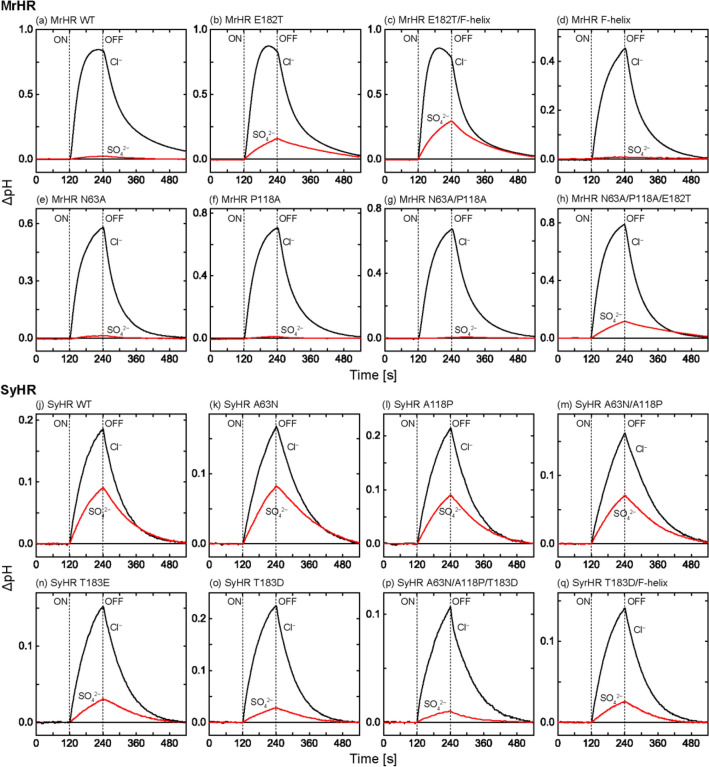
Anion pump activities of MrHR, SyHR and their mutants. The light-induced pH changes of *E. coli* suspensions were measured in the presence of 10 μM CCCP. The results for MrHR and the mutants are shown in the upper eight panels, and those for SyHR are shown in the lower panels. Each panel contains two time traces measured in the presence of 0.2 M NaCl (black line) and 0.2 M Na_2_SO_4_ (red line). The initial pH values were 6.0–6.2. The vertical lines indicate the timing of illumination, whose intensity at 530 nm was set to 27.8 mW/cm^2^.

We employed a pH electrode to examine the anion pumping activities of MrHR and SyHR. Their anion transport into the cell interior creates a negative membrane potential inside, which in turn drives passive H^+^ inflow across the cell membrane. Thus, the anion pumping activity can be detected by the light-induced pH increase of the *Escherichia coli* suspension. This method is very simple and has been conventionally utilized for the study of electrogenic transporters. However, the measured signal often includes a significant artifact, because the scattering light from the sample hits the Ag/AgCl electrodes inside the body and then distorts the output voltage. In this study, it was necessary to detect a tiny pH change because we aimed to find the mutation that confers the SO_4_^2−^ pumping ability on MrHR. A small pH signal should contain a relatively large artifact, which might lead to incorrect conclusions. Thus, we explored the method to reduce the artifact and finally found that an India ink can completely remove the artifact. Supplementary Fig. S1 shows the picture of the pH electrode before (1) and after (2) the replacement of the internal KCl solution by the India ink dissolving 3.3 M KCl. The India ink completely covered both the internal and reference Ag/AgCl electrodes inside the body. Supplementary Fig. S1 also compares the output of pH electrodes when the *E. coli* suspension was illuminated. These *E. coli* cells did not harbor the expression plasmid for microbial rhodopsin. The cell density and the light intensity were the same as those of the subsequent experiments in Fig. 2. As shown here, the output from the electrode with normal KCl involved a distinct light-induced artifact whose shape is quite similar to the pH change induced by true anion pumping activity. In contrast, the electrode with India ink was completely insensitive to light. This “black” electrode was employed for all following experiments.

### The key residue that confers SO_4_^2−^ pumping ability

All anion pumps are known to bind substrate anions in the vicinity of the protonated Schiff base in the dark state. This is also the case for SyHR, which can bind SO_4_^2−^ and other transportable anions^9^. In contrast, MrHR has no ability to bind SO_4_^2−^^8^. This difference might be a key determinant for the SO_4_^2−^ transport ability. Thus, we explored the differences in amino acid residues near the Schiff base between MrHR and SyHR. Supplementary Fig. S2 shows the residues of MrHR within 5 Å of the Schiff base nitrogen or the substrate Cl^−^. Their corresponding residues in SyHR are indicated in parentheses. As shown here, all 10 residues are shared between MrHR and SyHR. Thus, there are no characteristic differences in the Schiff base regions.

Next, we focused on the Glu182 residue of MrHR, which is located on the EC surface and corresponds to Thr183 of SyHR (Fig. 1a,b). Our previous study indicated that Glu182 of MrHR is deprotonated in the dark state and that the E182Q mutation elevates Cl^−^ pumping activity^12^. These results may indicate that the negative charge of Glu182 is located in the anion entrance pathway. Thus, we examined the mutation effect of Glu182 on the SO_4_^2−^ pumping ability. The results are shown in Fig. 2. For wild-type MrHR (Fig. 2a), the pH value with SO_4_^2−^ (red line) showed a slight upward shift upon illumination. As mentioned above, the India ink completely removes the light-induced artifact. Thus, MrHR seems to have a faint ability to pump SO_4_^2−^. In contrast, as shown in Fig. 2b, the E182T mutant exhibited distinct SO_4_^2−^ pumping activity.

**Figure 3:**
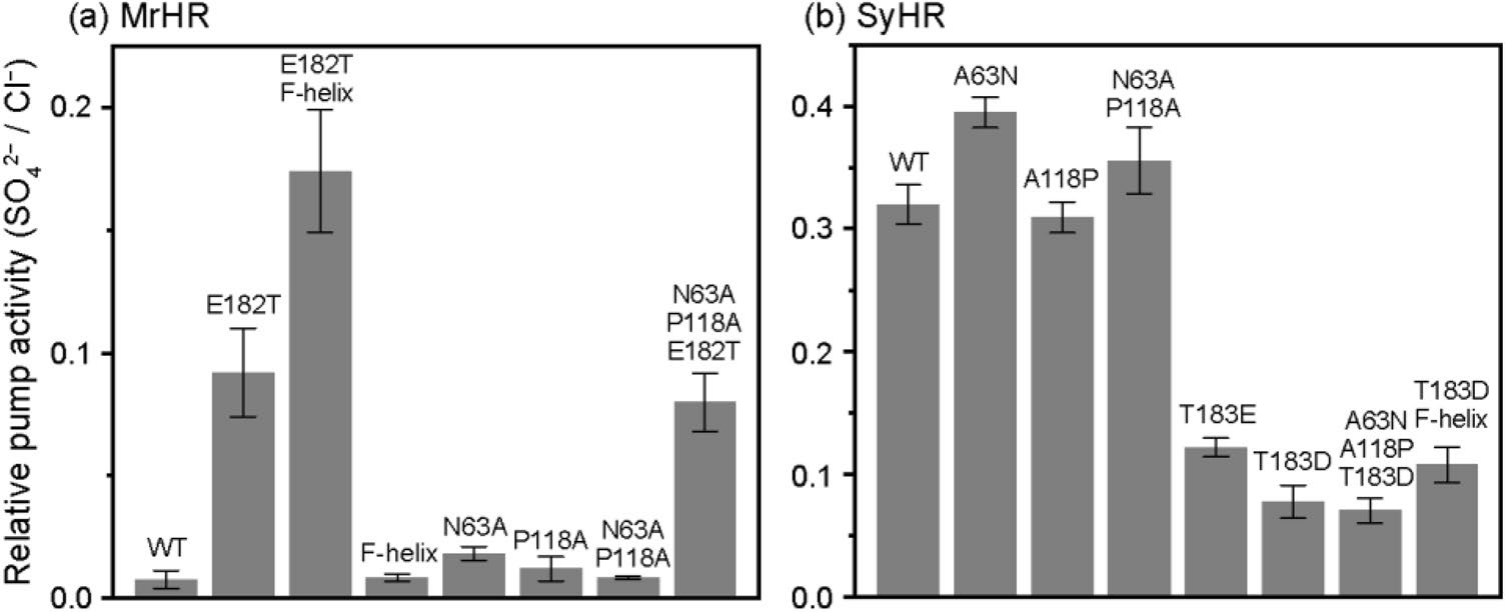
Relative SO_4_^2−^ pump activities with respect to the corresponding Cl^−^ pump activities. These values were calculated from the initial slopes of pH changes in Fig. 2 (for details, see text) and plotted in (**a**) for MrHR and the mutants and in (**b**) for SyHR. Each bar indicates the mean ± standard deviation (n = 3–5).

The initial slope of the light-induced pH change reflects the ion pump activity. In Supplementary Fig. S3, the slopes with Cl^−^ and SO_4_^2−^ are summarized after compensations using relative expression amounts of ion pumps, whose values are also plotted in the bottom panels. As shown in the upper four panels, the mutations affect not only the SO_4_^2−^ pump activity but also the Cl^−^ pump activity. Thus, to evaluate the extent to which each mutation elevated the activity for SO_4_^2−^ relative to that for Cl^−^, we also calculated the relative activity by dividing the slope for SO_4_^2−^ by the corresponding slope for Cl^−^. These values are plotted in Fig. 3 and are mainly referred to hereafter. These relative activities are independent of the expression amounts of ion pumps, because both samples for the measurements of SO_4_^2−^ and Cl^−^ pump activities were simultaneously prepared from the same *E. coli* culture medium. Thus, each sample surely contained the same amounts of proteins.

As shown in Fig. 3a, the relative pump activity of wild-type MrHR was 0.008, but this value was increased to 0.092 by the E182T mutation. Thus, this mutation strengthens SO_4_^2−^ pump activity by a factor of approximately 11. We also tested the replacement of Glu182 with Ser, Gln, Arg, Lys, and Leu. However, all these mutants exhibited almost the same relative pump activity as the E182T mutant (data not shown). As shown in Figs. 2c and 3a, further enhancement of the activity was achieved by additional mutation of the residues near the cytoplasmic (CP) end of the F-helix (blue sticks in Fig. 1a–c). In this region, there exists a characteristic difference in the residues between MrHR and SyHR, as previously pointed out by Niho et al.^9^. As shown in Fig. 1d, on the upstream side (CP end side), SyHR has more basic residues (red letters) than MrHR. Thus, we replaced the five residues of MrHR enclosed by dashed squares (Fig. 1d) with the corresponding residues from SyHR. This mutation itself, which will be referred to as the “F-helix”, had no effect on the SO_4_^2−^ pumping activity (Fig. 2d). However, the simultaneous mutation with E182T (E182T/F-helix) resulted in a distinct increase in this activity (Fig. 2c). The effect of the simultaneous mutation was also confirmed by the relative pump activity in Fig. 3a. Thus, Glu182 is a key residue. The removal of its negative charge seemed to be a prerequisite for the distinct SO_4_^2−^ pumping activity of MrHR.

**Figure 4:**
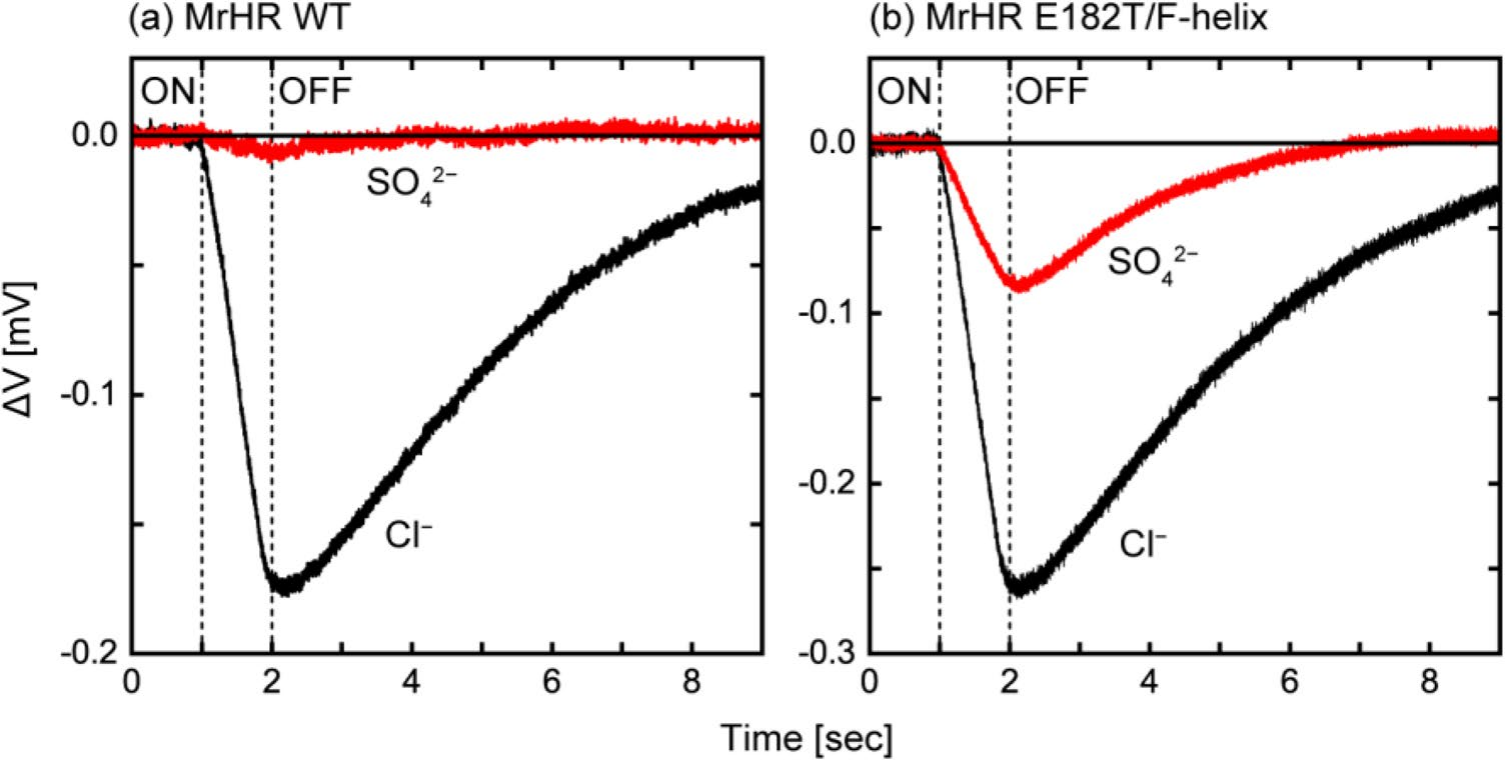
Anion pump activities detected by ITO electrode. The schematic illustration of the electrochemical cell is shown in Supplementary Fig. S4. The light-induced pH differences between the *E. coli* suspensions and the reference salt solutions were detected as the voltage difference between two ITO electrodes. Two time-courses of the voltage differences in the presence of 0.2 M NaCl (black line) and 0.2 M Na_2_SO_4_ (red line) were plotted for wild-type MrHR (**a**) and the E182T/F-helix mutant (**b**), respectively. All solutions also contained 10 μM CCCP. The vertical lines indicate the timing of illumination. The negative deflected change in the voltage difference corresponds to the pH increase.

The pH electrode contains 3.3 M KCl as internal solution, which slightly leaks into the sample medium. If the mutation significantly elevates the binding affinity for Cl^−^, the resultant mutant might cause a detectable pH change in the SO_4_^2−^ solution by transporting contaminating Cl^−^, even though it does not have the ability to pump SO_4_^2−^. To further confirm the SO_4_^2−^ pumping activity, we next used the “Cl^−^ free” method. Here, we employed an electrochemical cell using an indium-tin oxide (ITO) electrode, whose schematic structure is shown in Supplementary Fig. S4. ITO electrode is an ITO-coated transparent electrode and works as a pH sensor with rapid response^13^. As similar to the measurements in Fig. 2, anion pump activity elevates the pH of *E. coli* suspension (Supplementary Fig. S4). This pH increase was recorded as the voltage difference between two ITO electrodes (for details, see Materials and Methods). Here, we recorded the light-induced voltage change for a short period of time (9 s) with a 1 s illumination. This short-time measurement is possible because of the fast response of the ITO electrode and has the advantage that voltage baseline fluctuations can be ignored. The measured voltage changes are shown in Fig. 4. The negative voltage change corresponds to the alkalization of the medium. In the presence of Cl^−^, both of wild-type MrHR and its E182T/F-helix mutant caused distinct voltage changes (Fig. 4a,b, black lines). In contrast, in the presence of SO_4_^2−^, only the mutant MrHR caused distinct voltage change (red lines). Thus, this mutant surely has the ability to pump SO_4_^2−^. We also examined its affinity for Cl^−^ and compared it with that of wild-type MrHR. The results are summarized in Supplementary Fig. S5. The determined dissociation constants were 4.98 mM and 1.18 mM for wild-type and the mutant MrHR, respectively. Thus, this mutation indeed lowered the dissociation constant, but not to a remarkably strong value.

The tertiary structure of MrHR was solved by two groups^10,11^. Yun et al. also created mutants with SO_4_^2−^ pumping activity based on their determined structure^10^. In addition to Glu182 described above, they mutated the neighboring residues Asn63 and Pro118 (Fig. 1a,b), which correspond to Ala63 and Ala118 in SyHR, respectively. Upon replacement of these neighboring residues with their counterparts in SyHR (N63A/P118A), they observed the strongest SO_4_^2−^ pumping activity. Thus, to clarify the key determinant for SO_4_^2−^ pumping activity, we performed further mutation studies on MrHR. The results are shown in Figs. 2e–h and 3a. In our measurements, neither the single mutations N63A (Fig. 2e) and P118A (Fig. 2f) nor their simultaneous mutation (Fig. 2g) conferred SO_4_^2−^ pumping activity on MrHR. In contrast, an additional mutation of E182T, i.e., the triple mutant N63A/P118A/E182T (Figs. 2h, 3a), resulted in distinct SO_4_^2−^ pumping activity. Consistent results were also obtained for SyHR (Figs. 2j–q, 3b). Here, we examined the "reverse" mutation, which reduces SO_4_^2−^ pumping activity. As shown in Fig. 2j, wild-type SyHR showed strong SO_4_^2−^ pumping activity, which was not affected by the mutations A63N, A118P, and A63N/A118P (Fig. 2k–m). However, a distinct decrease in the activity was induced by the mutations T183E, T183D, and A63N/A118P/T183D (Figs. 2n–p, 3b). Thus, for both MrHR and SyHR, the negative charge at the anion entrance pathway (182nd position for MrHR and 183rd position for SyHR) is probably the critical determinants of the SO_4_^2−^ pumping ability. Unlike MrHR, the simultaneous mutation of T183D with “F-helix” did not further reduce the SO_4_^2−^ pumping activity (Fig. 2q). This result might reflect a structural difference between these anion pumps.

As mentioned above, Supplementary Fig. S3e,f (bottom panels) show the relative expression amounts of anion pumps. These values were determined from the magnitudes of the flash-induced absorbance changes, which reflect the amounts of proteins undergoing the photocycles (see “Materials and Methods”). The validity of this method was examined in two experiments and the results were summarized in the Supplementary Information.

### Unique features of the MrHR mutant with SO_4_^2−^ pumping activity

**Figure 5:**
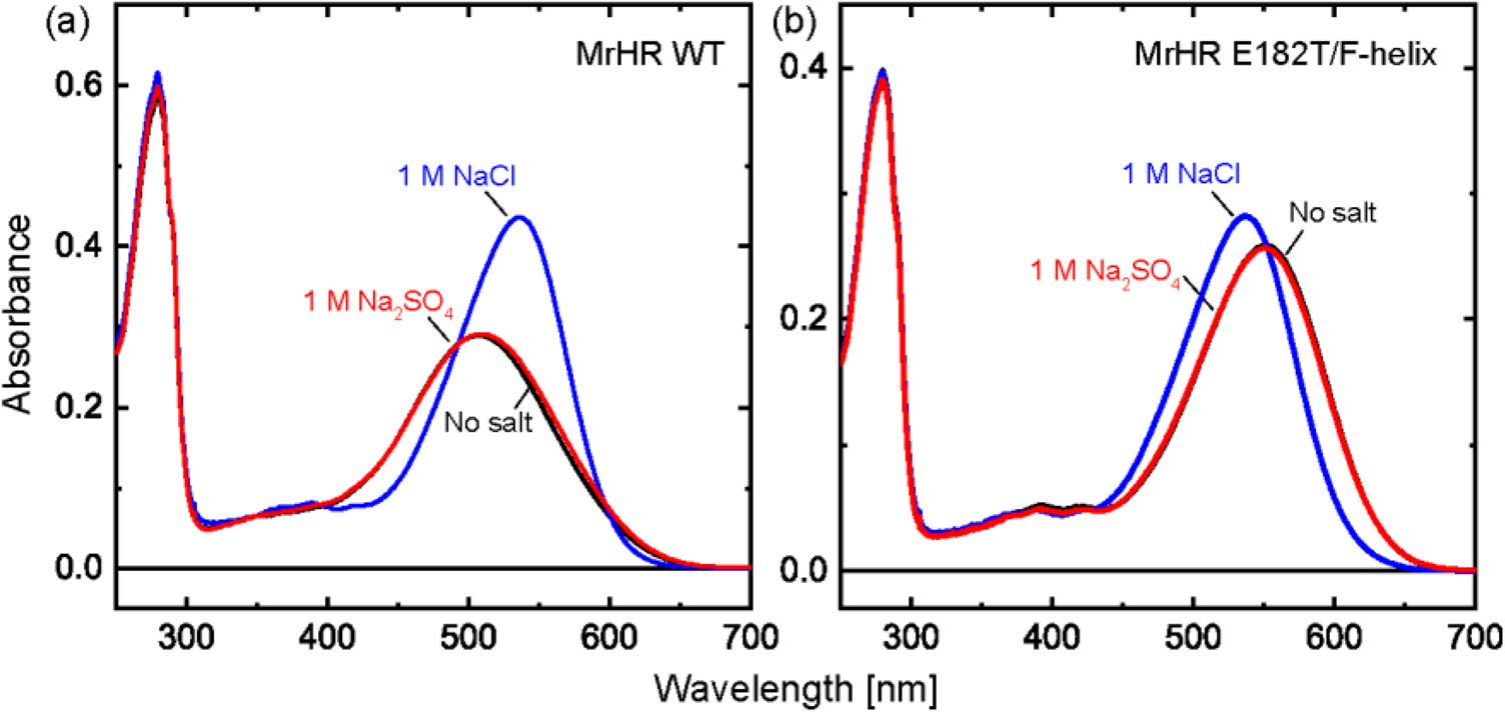
Absorption spectra of wild-type MrHR (**a**) and the E182T/F-helix mutant (**b**). The basal buffer was 50 mM citric acid, pH 6.0, containing 0.05% DDM.

This mutant should exhibit different features from the wild-type MrHR, reflecting the SO_4_^2−^ pumping ability. Thus, we performed basic characterizations of the E182T/F-helix mutant. However, all the results were unexpected. As mentioned above, unphotolyzed anion pumps already bind substrate anions in the vicinity of the protonated Schiff base. This is also the case for SO_4_^2−^ pumping SyHR^9^. The anion binding ordinarily induces shifts in the absorption spectra of the unphotolyzed states. However, this spectral shift was not observed for the E182T/F-helix mutant. Figure 5 shows the experimental results for the wild-type and the mutant. As mentioned above, wild-type MrHR does not bind SO_4_^2−^^8^, which was confirmed in Fig. 5a, where the absorption spectrum did not shift even when the concentration of SO_4_^2−^ was increased to 1 M (black and red lines). This result is in clear contrast to the significant redshift in the presence of 1 M Cl^−^ (blue line). MrHR binds Cl^−^ with the dissociation constant of 5 mM as described above (Supplementary Fig. S5). Thus, at 1 M Cl^−^, all proteins should bind Cl^−^. For the E182T/F-helix mutant, Cl^−^ binding was also observed, as shown in Fig. 5b (blue line). Here, the spectrum shifted to the shorter wavelength region, which is the opposite direction from that of wild-type MrHR. We previously reported this opposite shift for the E182Q single mutant and concluded that the original Glu182 residue in the wild-type MrHR is deprotonated and has a long-distance interaction with retinal chromophore^12^. Thus, this blueshift of the E182T/F-helix mutant was not surprising to us. An unexpected result was the “absence” of a spectral shift upon the addition of SO_4_^2−^ (red line). This result suggests that no SO_4_^2−^ binding occurs in the vicinity of the protonated Schiff base at 1 M SO_4_^2−^, even though we detected SO_4_^2−^ pumping activity at 0.2 M SO_4_^2−^. Thus, this mutant seemed to capture SO_4_^2−^ in the protein interior after light activation. The spectra in Fig. 5 were measured in the detergent-solubilized states of the proteins so that the light scattering artifact was small. Solubilization often distorts the properties of membrane proteins. Thus, we also examined the spectral shift of the membrane fragment of *E. coli* cells expressing MrHR. After disruption of the *E. coli* cells, the membrane fragments were collected and encapsulated in the acrylamide gel to avoid their precipitation. After replacement of the buffer solutions, their absorption spectra were measured. As shown in Supplementary Fig. S6, with the addition of Cl^−^, both the wild-type and mutant MrHR exhibited distinct spectral shifts, which were saturated in the presence of 0.2 M Cl^−^. In contrast, for SO_4_^2−^, no spectral shifts were observed at 0.2 M for both samples. Slight redshifts were detected at 1 M SO_4_^2−^ for both samples, but these shifts were faint compared to those upon Cl^−^ binding and might be caused by the significant increase in ionic strength. Thus, SO_4_^2−^ binding does not seem to occur regardless of detergent-solubilized and unsolubilized states.

**Figure 6:**
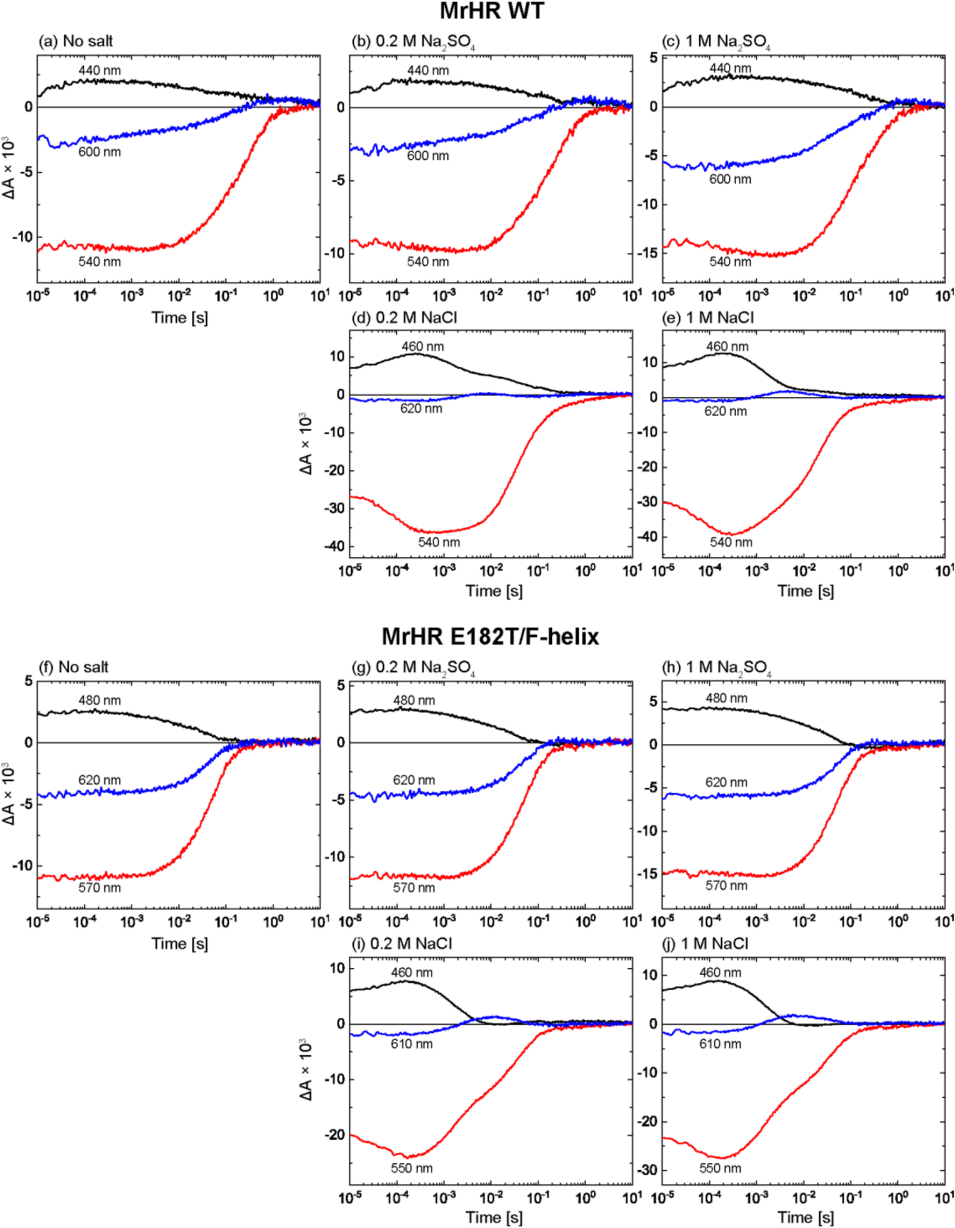
Flash-induced absorbance changes of wild-type MrHR (**a**–**e**) and the E182T/F-helix mutant (**f**–**j**). The *E. coli* membrane fragments were used for the samples after encapsulation into 15% acrylamide gels. The buffer solutions were 50 mM citric acid, pH 6.0, containing the salts indicated above the respective panels.

Next, we examined the photoreactions by measuring the flash-induced absorbance changes and obtained further unexpected results for the mutant. Here, we used *E. coli* membrane fragments for the samples to prevent detergent-induced artifacts. However, the results were essentially the same as those for the detergent-solubilized state and the lipid-reconstituted state. Figure 6 compares the photocycles under no salt conditions and under conditions with SO_4_^2−^ or Cl^−^. The corresponding difference spectra are summarized in Supplementary Fig. S7. As shown in Fig. 6a, wild-type MrHR exhibited a relatively simple photoreaction in the absence of salt, where a short-wavelength intermediate appeared at approximately 440 nm in the early time range. With its concomitant decay, the last intermediate appeared at approximately 600 nm and then decayed with a slow lifetime of approximately several seconds. This "nonpumping photocycle" was still observed in the presence of 0.2 M and 1 M SO_4_^2−^ (Fig. 6b,c), reflecting the lack of SO_4_^2−^ pumping activity of the wild-type MrHR. In contrast, in the presence of 0.2 M and 1 M Cl^−^ (Fig. 6d,e), a significantly different photocycle, i.e., the “Cl^−^ pumping photocycle”, was observed, where several intermediates sequentially appeared. They were previously assigned to initial K at 540 nm, L at 460 nm at approximately 0.3 ms, and the mixture of N at 460 nm and O at 620 nm at approximately 10 ms, respectively^12^. Finally, the slight negative absorption change at 540 nm, which was attributed to the accumulation of the last intermediate MrHR', remained for several seconds. Essentially, the same “Cl^−^ pumping photocycle” was observed for the E182T/F-helix mutant in the presence of 0.2 M and 1 M Cl^−^ (Fig. 6i,j), reflecting the conserved Cl^−^ pumping activity of this mutant. On the other hand, in the no salt condition (Fig. 6f), this mutant exhibited a simple photocycle, which is similar to the corresponding photocycle of the wild-type MrHR. The initial intermediate at 480 nm probably corresponds to the 440 nm intermediate for the wild-type MrHR (Fig. 6a). The difference in the observed wavelength reflects the 42 nm redshift of the unphotolyzed state spectrum compared to that of the wild-type MrHR (Fig. 5 and Supplementary Fig. S6). The distinct difference from the wild-type MrHR is faster termination of the photoreaction and the negligible accumulation of the long-wavelength intermediate. These differences seem to be reasonable because the E182T/F-helix mutation confers SO_4_^2−^ pumping ability and thus should have a significant impact on the protein. However, the photocycles in the presence of SO_4_^2−^ were unexpected (Fig. 6g,h). Similar to the case in the wild-type MrHR, the photocycle in the no salt condition (Fig. 6f) was still observed even in the presence of 0.2 M and 1 M SO_4_^2−^ (Fig. 6g,h). As mentioned above, this mutant might capture SO_4_^2−^ after starting the photoreaction. Even in this case, we expected that the photocycle would change upon the addition of SO_4_^2−^, reflecting the SO_4_^2−^ pumping reaction. Thus, the photocycles with SO_4_^2−^ (Fig. 6g,h) were unexpected but might be explained by considering several assumptions. Possible explanations will be discussed later.

### Unique features of SyHR mutant with reduced SO_4_^2−^ pumping activity

**Figure 7:**
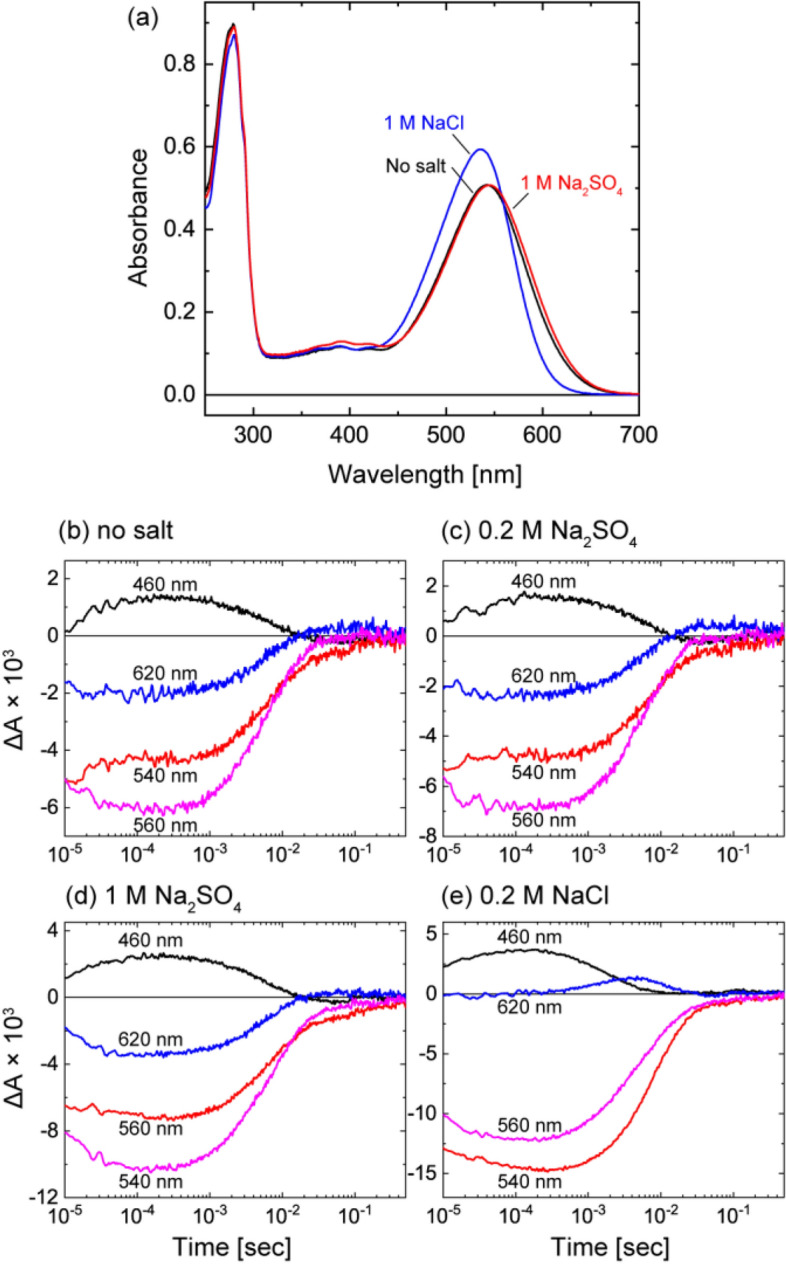
Absorption spectra (**a**) and photoreactions (**b**–**e**) of T183D SyHR. The basal buffer was 50 mM citric acid, pH 6.0. (**a**) The purified protein was used. All buffer solutions contained 0.05% DDM. (**b**–**d**) The sample preparations and the measuring conditions were the same as those of Fig. 6.

The T183D mutation clearly reduced the SO_4_^2−^ pump activity. Thus, this mutant should exhibit different characteristics from the wild-type SyHR. One of the differences might be the lower affinity for SO_4_^2−^, because the wild-type MrHR does not have SO_4_^2−^ pumping activity and does not bind SO_4_^2−^. In contrast, the wild-type SyHR binds SO_4_^2−^ with a dissociation constant of 5.8 mM dissociation constant, as determined by a distinct redshift in the absorption spectrum^9^. Thus, we examined the spectral shift of SyHR T183D mutant. The results are shown in Fig. 7a. Upon addition of Cl^−^ (blue line), this mutant showed distinct spectral shift. In contrast, only faint shift was observed even in the presence of 1 M SO_4_^2−^ (red line), so that we could not determine the dissociation constant. For this mutant, we also examined the SO_4_^2−^-induced change in the photoreaction. The time-courses of flash-induced absorbance changes at typical four wavelengths are shown in Fig. 7b–e, and the corresponding difference spectra are shown in Supplementary Fig. S8. As described above, the T183D SyHR still maintained 24% of the SO_4_^2−^ pump activity of wild-type SyHR (Fig. 3b). However, even in the presence of 0.2 M and 1 M SO_4_^2−^ (Fig. 7c,d), this mutant showed almost the same photoreaction as under no salt condition (Fig. 7b). These results are clear contrast to the distinct changes in the photoreaction after addition of Cl^−^ (Fig. 7e). Thus, this SyHR mutant might have the features similar to those of the E182T/F-helix MrHR. Both mutants might start photoreaction without the SO_4_^2−^ binding in the vicinity of the retinal but can transport it in a unique mode. In this study, we aimed to convert MrHR into SyHR. But, the resultant E182T/F-helix MrHR is not comparable to SyHR. This mutant might be an intermediate between MrHR and SyHR, and comparable to the T183D SyHR mutant.

## Discussion

In this study, we aimed to clarify the determinant for SO_4_^2−^ pumping ability on the cyanobacterial Cl^−^ pump. By the studies using various mutants, we concluded that the negative charge at the anion entrance gate is an important “switch” for the SO_4_^2−^ pumping ability. For MrHR, the removal of the negative charge is a prerequisite to transport SO_4_^2−^ (Figs. 2a–h, 3a). In other words, the transport ion is determined by its entrance and not by the “central” retinal Schiff base region. The reverse mutations on SyHR, i.e., the introductions of acidic residues to the same position clearly weakened the SO_4_^2−^ pumping activity (Figs. 2n–q, 3b). However, in contrast to MrHR, distinct SO_4_^2−^ pumping activity remained for the SyHR mutants. This weaker effect of mutation might reflect the structural difference between MrHR and SyHR. For SyHR, the introduced acidic residues might not be fully deprotonated, and/or the entrance gate might not be tight and then fail to completely expel SO_4_^2−^, even in the presence of a negative charge. The difference between MrHR and SyHR was also observed in the mutation effects of the “F-helix”. This region of SyHR has more basic residues than that in MrHR (Fig. 1d). By replacing the five residues of this region, the SO_4_^2−^ pumping activity of the MrHR mutant was further enhanced (Fig. 1h). Based on the MrHR structure (Fig. 1c), the introduced basic residues do not seem to interact with SO_4_^2−^, because those residues probably face the outside of the protein. However, their positive charges might contribute to SO_4_^2−^ transport through modulation of the dipole moment. The F-helix spans the membrane from the CP to EC side and thus has a dipole moment that facilitates anion movement toward the CP side. The introduced positive charges might enhance the dipole moment and then facilitate the release of SO_4_^2−^ through the pathway along the F-helix. In contrast, the counter mutation in SyHR (Fig. 2q), i.e., the replacement of the five residues with those of MrHR did not lower the SO_4_^2−^ pumping activity. Thus, the CP end structures of the F-helices might also be different between MrHR and SyHR.

Our experimental results disagree with those of Yun et al. in the following two respects^10^: the mutation to confer SO_4_^2−^ pumping activity on MrHR and the SO_4_^2−^ binding affinity of the mutant. As mentioned above, we detected the SO_4_^2−^ pumping activity of MrHR only when Glu182 was replaced by a non-acidic residue. Moreover, the resultant SO_4_^2−^ pumping MrHR mutant (E182T/F-helix) did not exhibit an absorption spectral shift even in the presence of 1 M SO_4_^2−^. Yun et al.^10^ observed SO_4_^2−^ pumping activity upon not only the replacement of Glu182 but also the replacement of Asn63 and its simultaneous replacement with Pro118. The latter two residues, Asn63 and Pro118, are located in the vicinity of Glu182 (Fig. 1a,b). Thus, their mutations might affect the pKa of Glu182. If the mutations increase the pKa, Glu182 might protonate at a relatively low pH and then allow SO_4_^2−^ transport. Thus, mutations in the neighboring residues might indirectly lead to SO_4_^2−^ pumping activity. Concerning the SO_4_^2−^ binding affinity, Yun et al.^10^ detected SO_4_^2−^-induced absorbance changes at approximately 530 nm for many mutants. However, in most cases, the absorbances at approximately 400 nm simultaneously changed, implying that the absorbance change at approximately 530 nm might be caused by the change in the protonation state of the Schiff base and might not reflect the binding of SO_4_^2−^. SO_4_^2−^ binding should cause not only an absorbance change but also a spectral shift. However, they detected only faint spectral shifts for many mutants. For the E182T/F-helix MrHR mutant (Fig. 5b), the measured spectra were superimposed over a wide wavelength region regardless of the absence or presence of SO_4_^2−^.

All other anion-pumping rhodopsins are known to bind substrate anions in the vicinity of the protonated Schiff base. Upon illumination, the retinal undergoes isomerization from the all-*trans* to the 13-*cis* form, which flips the orientation of the Schiff base NH^+^ dipole moment from the EC to the CP side. Thus, the initial anion binding seems reasonable for the orientation change of the dipole moment to be effectively utilized for anion movement from the EC side to the CP side. The anion binding induces a shift in the absorption spectrum for all other anion pumps^8,9,14–16^. Thus, the absence of a spectral shift for the E182T/F-helix MrHR was unexpected. We initially thought that this result was an artifact due to the distorted protein structure in the detergent solubilization state. However, essentially the same results were obtained even in the *E. coli* membrane fragments. Thus, SO_4_^2−^ surely induces no spectral shift for this mutant. Similar result was also observed for the T183D SyHR. This SyHR mutant maintained small SO_4_^2−^ pumping activity. However, only negligible spectral shift was observed even in the presence of 1 M SO_4_^2−^. These results suggest the following two possibilities: (1) SO_4_^2−^ binds to the site far from the Schiff base and thus does not induce the spectral shift, or (2) SO_4_^2−^ is captured after starting the photoreaction and then released before recovery to the original state. The sulfate might bind to the protein interior in the forms of HSO_4_^−^ or H_2_SO_4_. Even in this case, their binding likely alters the electrostatic environment and/or the surrounding structure. Thus, the absence of spectral change probably reflects no binding of the substrate near the protonated Schiff base. In both possibilities described above, SO_4_^2−^ comes to the Schiff base region after starting the photoreactions.

Substrate uptake in the photolyzed state is known to occur in Na^+^-pumping rhodopsin^17^, which captures Na^+^ during O intermediate formation and then releases it during O decay^18,19^. The MrHR and SyHR mutants might transport SO_4_^2−^ with a mode similar to that of the Na^+^ pump. Even in this case, it is reasonable to consider that the SO_4_^2−^ pumping photocycle is different from the nonpumping photocycle. However, we did not detect a distinct difference between them (Figs. 6f–h, 7b–d). One possible explanation for these results might be a two-photon reaction. Here, we used a short laser pulse to examine the photoreaction, whereas the ion pumping activity was detected under continuous illumination. If SO_4_^2−^ transport might be driven by the light excitation of a certain intermediate, the SO_4_^2−^ pumping reaction could not be detected by flash photolysis experiments. Thus, we tried to show this possibility but failed. Supplementary Fig. S9 summarizes the light-intensity dependence of the SO_4_^2−^ pumping activity of the MrHR mutant. The left panel shows the time-dependent pH changes induced by SO_4_^2−^ transport at various illumination intensities. The initial slopes of the pH changes are also plotted in the right panel against the illumination intensities. If two-photon excitation is a prerequisite for the SO_4_^2−^ pumping reaction, the activity becomes stronger as the intensity increases. Thus, in the simple case, there would appear to be a lag phase in the right panel at low illumination intensity, and the plot has a sigmoidal shape. However, such a tendency was not observed. The plot has no lag phase and shows simple saturation behavior, which is typical of a one-photon process. Thus, we failed to show the possibility of a two-photon process. The other possibility is that a small amount of protein undergoes the SO_4_^2−^ pumping photocycle. For E182T/F-helix MrHR, the activity of SO_4_^2−^ transport was 17.4% of the Cl^−^ transport activity (Fig. 3a). The corresponding value for T183D SyHR was 7.8% (Fig. 3b). The light-induced pH change reflects passive H^+^ inflow in response to the interior negative membrane potential created by inward anion pump activity. If sulfate is transported in the divalent SO_4_^2−^ form, a twofold larger membrane potential is created compared to the same amount of Cl^−^ translocation. Thus, for the activities of the mutants, only 8.7% (E182T/F-helix MrHR) and 3.9% (T183D SyHR) of proteins might undergo the SO_4_^2−^ pumping photocycles. Consequently, most proteins might still undergo respective nonpumping photocycles in the presence of SO_4_^2−^. However, even in this case, some difference would be likely to appear when the concentration of SO_4_^2−^ is increased from 0.2 to 1 M (Figs. 6g,h, 7c,d). Thus, there might exist a mechanism that keeps the amount of SO_4_^2−^ pumping protein low even at high concentrations of SO_4_^2−^.

In this study, we developed the pH electrode method by utilizing India ink as an internal KCl solution and removed light-induced artifacts. By using this method, we clarified that the absence of the negative charge at the anion entrance is important for SO_4_^2−^ pumping activity. This conclusion was further confirmed by the activity measurements using ITO electrode and the reverse mutation in SyHR. However, the subsequent characterizations of the resultant MrHR and SyHR mutants provided unexpected results. Unlike other anion pumps, these mutants seem to capture SO_4_^2−^ to the Schiff base region after starting the photoreaction. This photoreaction should be different from that of respective nonpumping reactions, but such a difference was not detected even at high concentrations of SO_4_^2−^. Thus, although uncertainty still remains, these mutants probably pump SO_4_^2−^ with a unique mode that has not been reported for anion pump rhodopsins. The detailed characterization of this translocation mode should be an interesting subject for future investigation.

## Materials and methods

### Protein expression and purification

*E. coli* DH5α was used for DNA manipulation. The expression plasmids of MrHR and SyHR, which were encoded in the pET vector, have been reported previously^8,9^. Both plasmids resulted in proteins having a six-histidine tag in the C-terminus. All single amino acid mutations were introduced using the QuikChange Site-Directed Mutagenesis kit (Agilent Technologies). For the replacement of five consecutive residues, the inverse PCR method was used. The DNA sequences were confirmed by a standard procedure.

For expression and purification, *E. coli* strains BL21(DE3) and C43(DE3) were used for MrHR and SyHR, respectively. For SyHR, BL21(DE3) expressed a faint amount of protein, but this expression level was greatly improved by C43(DE3). The procedures were essentially the same as those previously described^20,21^. Briefly, the cells were grown in 2 × YT medium at 37 °C, and expression was induced by the addition of 1 mM isopropyl-β-D-thiogalactoside in the presence of 10 μM all-*trans* retinal. After 3–4 h of induction, the cells were harvested and then disrupted by sonication. The membrane fragments were collected by ultracentrifugation and solubilized by 1.5% n-dodecyl β-D-maltopyranoside (DDM). After removal of the insoluble fractions, the solubilized proteins were purified using nickel-nitrilotriacetic acid agarose. The buffer solution was replaced with the desired buffers by two passages over Sephadex G-25 in a PD-10 column (Amersham Bioscience).

### Spectroscopic measurements

The absorption spectra were measured by a UV1800 spectrometer (Shimadzu, Kyoto, Japan). In addition to the DDM-solubilized states of the purified proteins, fragments of *E. coli* cell membranes were also used as samples. The membrane fragments were prepared as follows. After disruption of *E. coli* cells, the membrane fragments were collected by ultracentrifugation. These fragments were resuspended in aqueous solution and then encapsulated into 15% acrylamide gel to prevent precipitation. To replace the solution, the gels were immersed several times in the desired buffer solutions at appropriate intervals. All spectral measurements were performed at room temperature.

These gels were also used for the measurements of flash-induced absorbance changes. In this method, the samples were illuminated by a second harmonic Nd-YAG laser (532 nm, 7 ns, 2 mJ). The subsequent absorbance changes were recorded with a single wavelength kinetic system. The details of the apparatus were described previously^22^. To improve the S/N ratio, 30 laser pulses were used at selected wavelengths. All measurements were performed at 25 °C.

### Anion pumping activity measurement by using pH electrode

The Cl^−^ and SO_4_^2−^ pumping activities were evaluated by a conventional pH electrode method. Suspensions of *E. coli* cells expressing anion pumps were prepared, and then their light-induced pH changes were measured. These suspensions were prepared as described previously^12,23^. Briefly, after 3–4 h of induction of protein expression, the culture was divided into two centrifugal tubes. The *E. coli* cells were harvested by centrifugation and washed twice with 0.2 M NaCl or 0.2 M SO_4_^2−^. The cells were suspended again with the same solutions and then gently shaken overnight at 4 °C in the presence of 10 μM carbonyl cyanide m-chlorophenylhydrazone (CCCP). The next day, these cells were further washed twice with the same solutions. Finally, the cells were suspended in a 10 ml volume containing 10 μM CCCP. The cell densities were adjusted so that the absorbance values at 660 nm became 2.0. The light source was a green LED light of 530 ± 17.5 nm (LXHL-LM5C; Philips Lumileds Lighting Co.), whose intensity was adjusted to the appropriate values at 530 nm by using an optical power meter (Orion-PD; Ophir Optronics Ltd). All measurements were performed at room temperature (approximately 25 °C).

The anion pump activities were quantified by the initial slopes of the pH increases. These activities depend on the protein expression levels. Thus, we also estimated these levels by the magnitudes of the flash-induced absorbance changes as previously described^12,22^. Briefly, after the activity measurements, the *E. coli* cells were collected by centrifugation and then resuspended in 2 mL of 50 mM MES, pH 6, containing 0.2 M NaCl. The cells were disrupted by sonication, and the resultant lysates were used for the measurements of flash-induced absorbance changes. The negatively deflected signals at 540 nm reached a maximum during 0.1–1 ms after flash illumination. These maximum values were used to estimate the expression levels. To compensate for the differences in the expression levels, the initial slopes of pH changes were divided by the respective maximum values of the flash-induced absorbance changes.

### Anion pumping activity measurement by using ITO electrode

The schematic illustration of the electrochemical cell is shown in Supplementary Fig. S4. The cell structure is essentially the same with those used in the previous study^24^. This cell consists of two ITO electrodes and a dialysis membrane which separates the *E. coli* suspension and the counter aqueous solution containing the same salt with the *E. coli* suspension (0.2 M NaCl or 0.2 M Na_2_SO_4_). Both solutions additionally contained 10 µM CCCP. The *E. coli* suspensions were prepared with the same procedure for the measurement by pH electrode, but the final cell densities were adjusted so that the absorbance values at 660 nm became 10.0. The ITO electrode is a transparent and senses the pH with rapid response. The anion pump activities elevate the pH of *E. coli* suspension compared to the counter solution. This pH difference was recorded as the voltage difference between two ITO electrodes. The *E. coli* suspensions were illuminated during 1 s, whose duration was controlled by a mechanical shutter. The voltage change was averaged for 50–60 times to improve the S/N ration. The light source was a 150 W xenon arc lamp in combination with two glass filters (IRA-25S, Y-50; Toshiba, Tokyo, Japan). The light intensity was 28.4 mW/cm^2^ at 530 nm, which was measured by the optical power meter described above. All measurements were performed at room temperature (approximately 25 °C).

## Supplementary Information

Supplementary Information.

## Data Availability

All data are available from the corresponding author upon reasonable request.
