# Supplementary material for: Mutations conferring SO42− pumping ability on the cyanobacterial anion pump rhodopsin and the resultant unique features of the mutant
Source: Sci Rep. 2022 Sep 30;12:16422. doi: 10.1038/s41598-022-20784-6 (PMC9525653; doi:10.1038/s41598-022-20784-6)
Supplement: Supplementary file 1 — Supplementary Information. [file 41598_2022_20784_MOESM1_ESM.pdf]

Supplementary information

Mutations conferring SO_4^{2-} pumping ability on the cyanobacterial anion pump rhodopsin and the resultant unique features of the mutant

Yuhei Doi[1], Jo Watanabe[1], Ryota Nii[1], Takashi Tsukamoto[1,2,3], Makoto Demura[1,2,3], Yuki Sudo[4], Takashi Kikukawa[1,2,3,*]

[1]School of Science, [2]Graduate School of Life Science, [3]Faculty of Advanced Life Science, Hokkaido University, Sapporo 060-0810, Japan. [4]Graduate School of Medicine, Dentistry and Pharmaceutical Sciences, Okayama University, Okayama 700-8530, Japan.

*Corresponding Author
Tel.: +81-11-706-3435
E-mail address: kikukawa@sci.hokudai.ac.jp
Postal address: Faculty of Advanced Life Science, Hokkaido University, Kita 10, Nishi 8, Kita-ku, Sapporo 060-0810, Japan

This file includes:

1. Supplementary figures
2. Validity of the method for estimating the Cl^- pump amounts in *E. coli* cells.

1. Supplementary figures

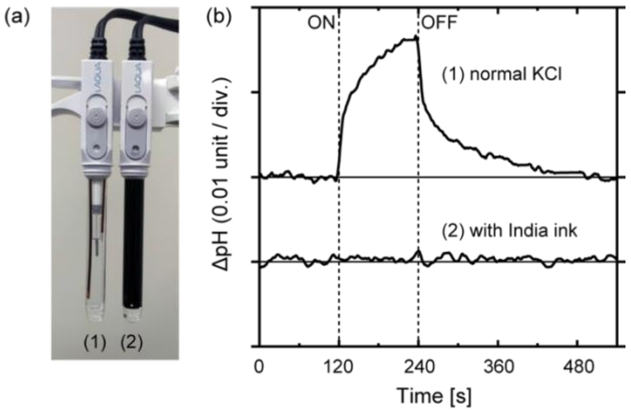

Figure S1. Removal of light-induced artifacts from the output of the pH electrode. (a) Photograph of pH electrodes before (1) and after (2) the replacement of internal KCl solutions with an India ink dissolved in 3.3 M KCl. (b) The light-induced artifact on the output of the pH electrode. The vertical broken lines indicate the illumination timing. Light-induced artifacts appeared for the electrode with normal KCl solution (1) but disappeared for that with India ink (2). The measuring conditions were the same as those for Fig. 2 except for the use of *E. coli* cells not harboring the expression plasmid of the anion pump.

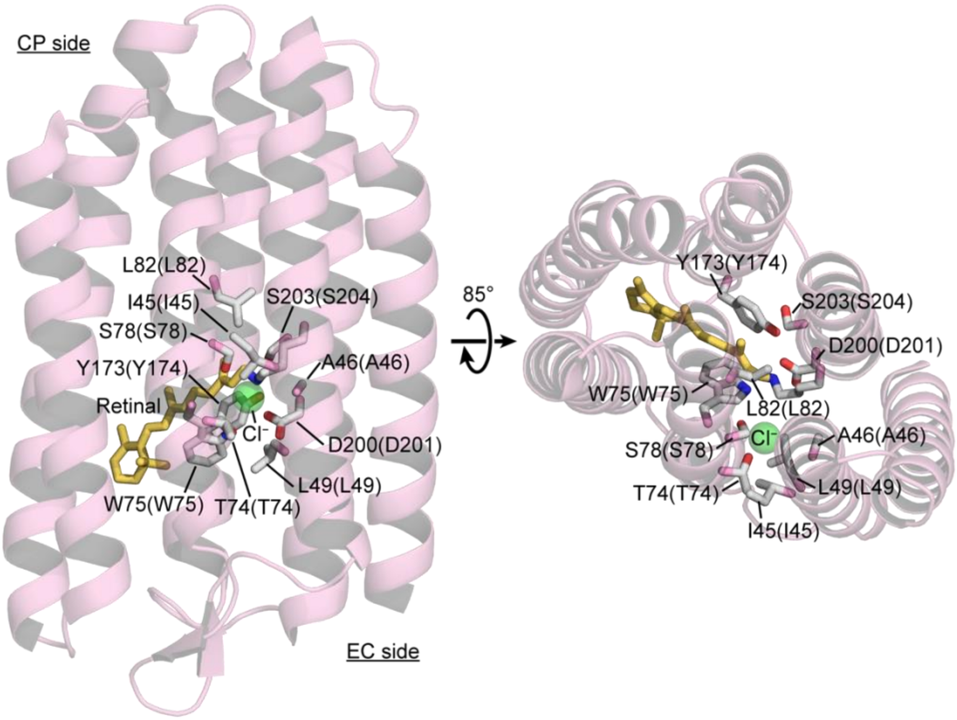

Figure S2. Amino acid residues located in the central part of MrHR. The residues within 5 Å either from the Schiff base nitrogen or the Cl^- ion are shown with a stick model. The corresponding residues in SyHR are indicated in parentheses. The PDB code is 6K6I.

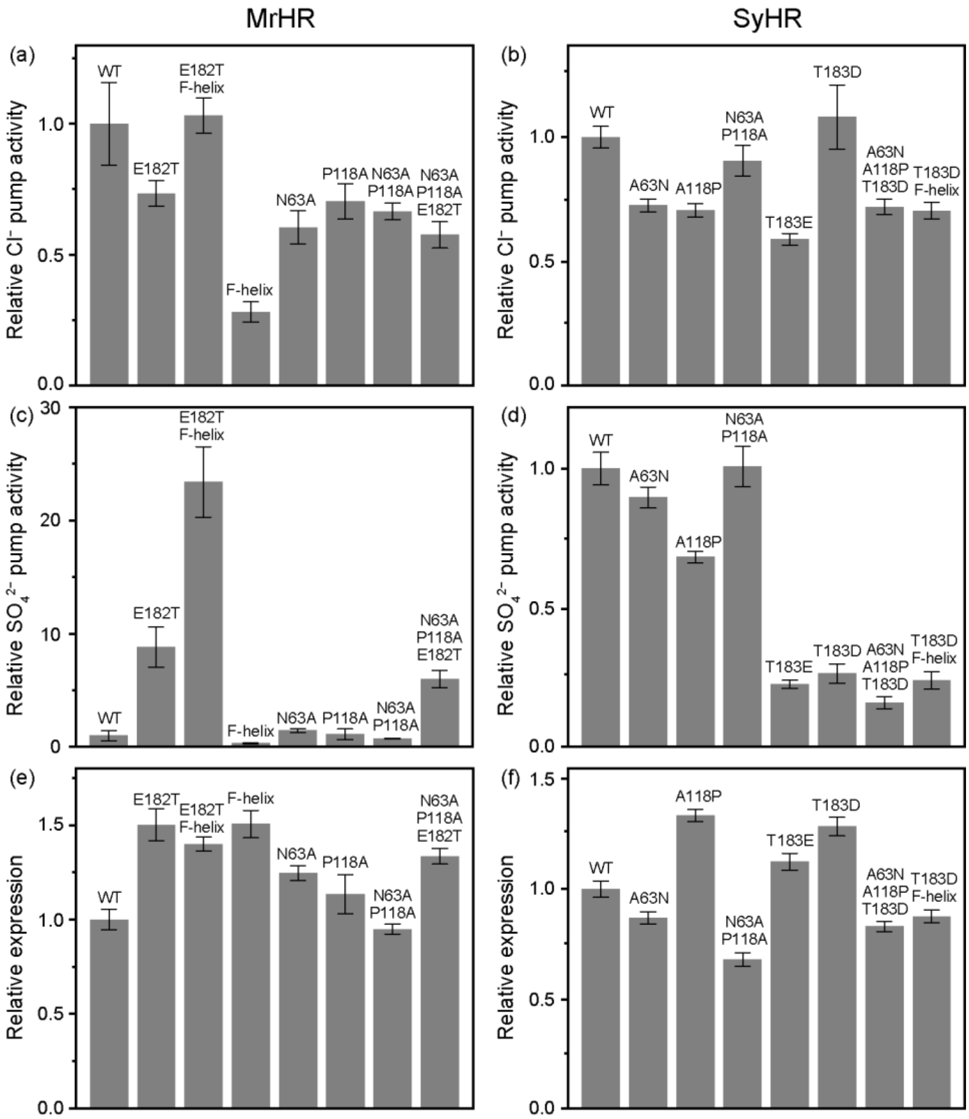

Figure S3. Cl^- and SO_4^{2-} pumping activities of MrHR, SyHR and their mutants. The initial slopes of pH changes in Fig. 2 were normalized by the respective expression levels of the anion pumps. The resultant relative values are plotted in the top (a, b) and middle (c, d) panels. The bottom panels (e, f) show the relative values of the expression levels, which were estimated from the maximum values of the flash-induced absorbance changes at 540 nm. Each bar indicates the mean ± standard deviation (n = 3–5).

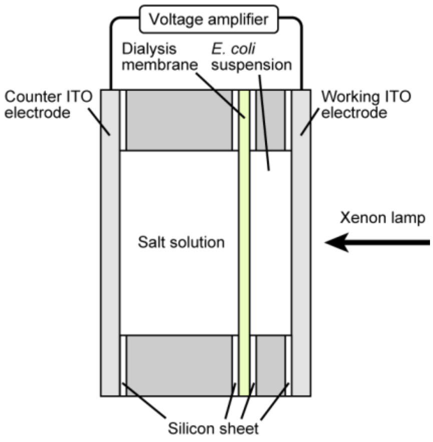

Figure S4. Schematic illustration of an electrochemical cell using ITO electrodes. The working electrode detects the pH of *E. coli* suspension, and the counter electrode detects the pH of a reference solution containing the same salt as the *E. coli* suspension. The two solutions are separated by a dialysis membrane, which electrically connects two solutions and prevents *E. coli* cells from leaking into the reference solution. The anion pump rhodopsin creates a pH difference between two solutions. This difference is detected as a voltage difference between the two ITO electrodes.

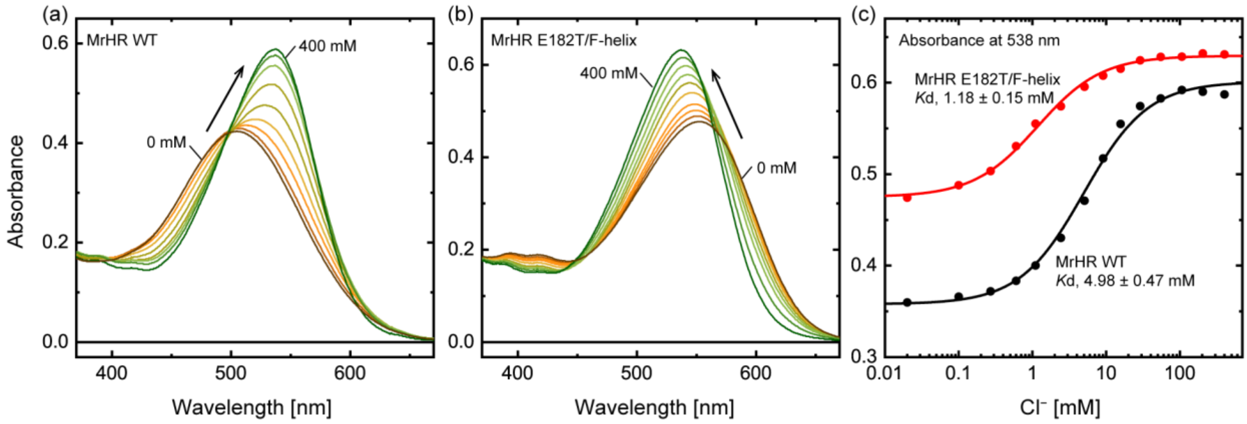

Figure S5. Cl^--induced absorption spectral shifts of the wild-type MrHR (a) and the E182T/F-helix mutant (b). The medium contained 50 mM citric acid (pH 6.0), 0.1% DDM and NaCl (0 – 400 mM). The shift directions are indicated with black arrows. The absorbance changes at 538 nm (A_{538}) were plotted in (c) and were fitted with the following equation:

$$A_{538} = \frac{[Cl^-]}{K_d + [Cl^-]} + y_0$$

where K_d, $[Cl^-]$, and y_0 represent the dissociation constant, Cl^- concentration, and the absorbance value in the absence of Cl^-, respectively. The determined K_d values are indicated in the panel.

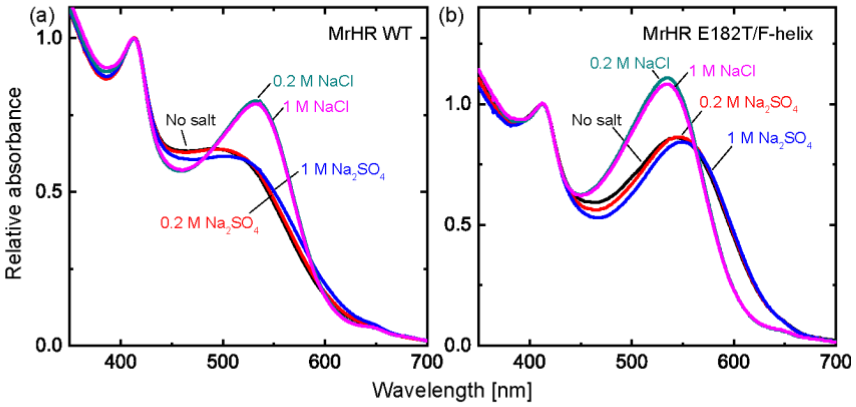

Figure S6. Absorption spectra of the *E. coli* membrane fragments containing wild-type MrHR (a) and the E182T/F-helix mutant (b). The basal buffer was 50 mM citric acid, pH 6.0. In addition to the bands at approximately 500–600 nm, narrow bands from cytochrome appeared at approximately 410 nm. The latter peak values were used to normalize the respective spectra.

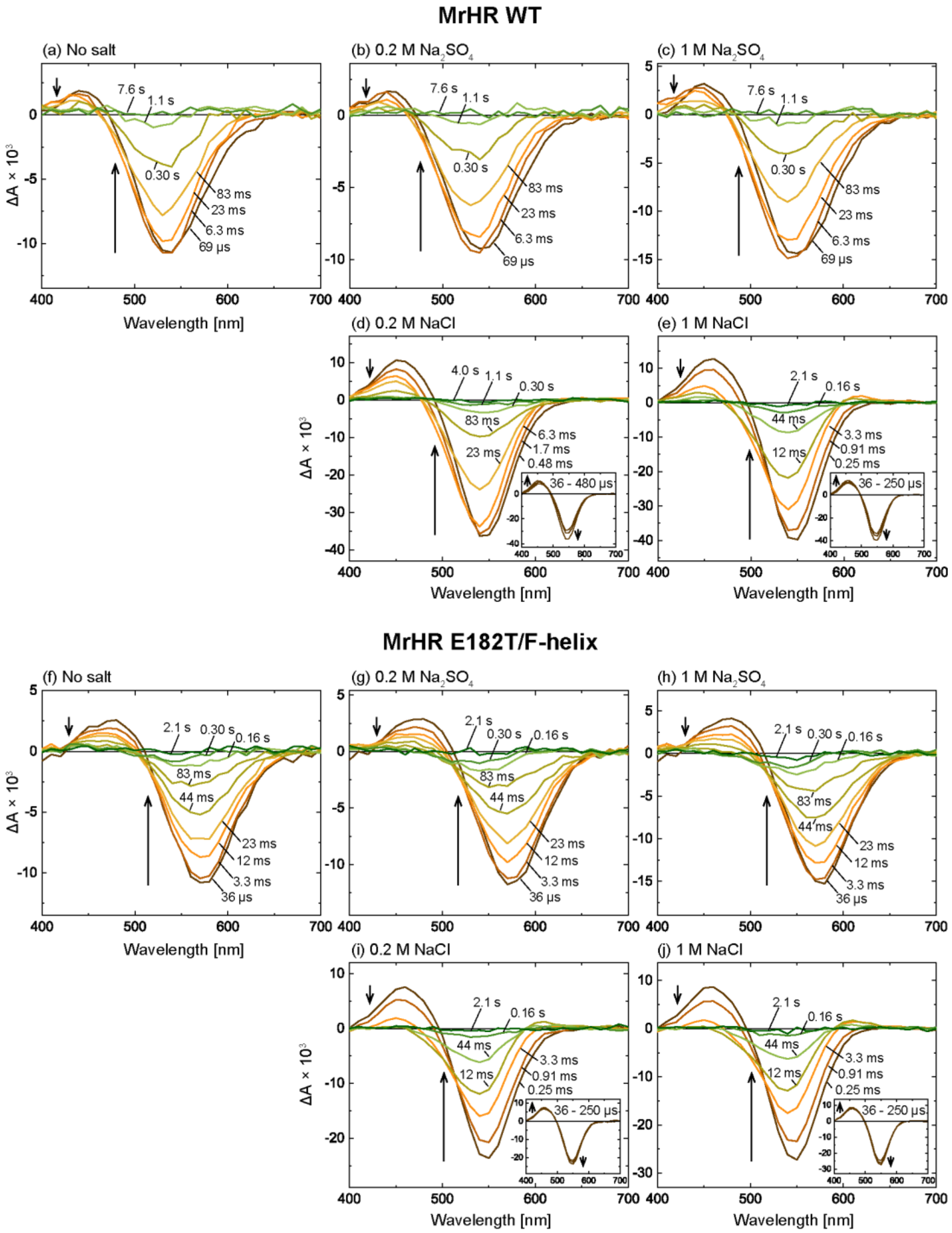

Figure S7. Light minus dark difference spectra of wild-type MrHR (a-e) and the E182T/F-helix mutant (f-j). The flash-induced absorbance changes were measured at 400-700 nm with 10 nm intervals. These data were used to calculate the difference spectra. The samples and the measuring conditions were the same as those in Fig. 6. In the presence of Cl^- (d, e, i, j), both proteins exhibited small spectral changes in the early time range (< 500 μs). These changes are plotted in the insets of the respective panels.

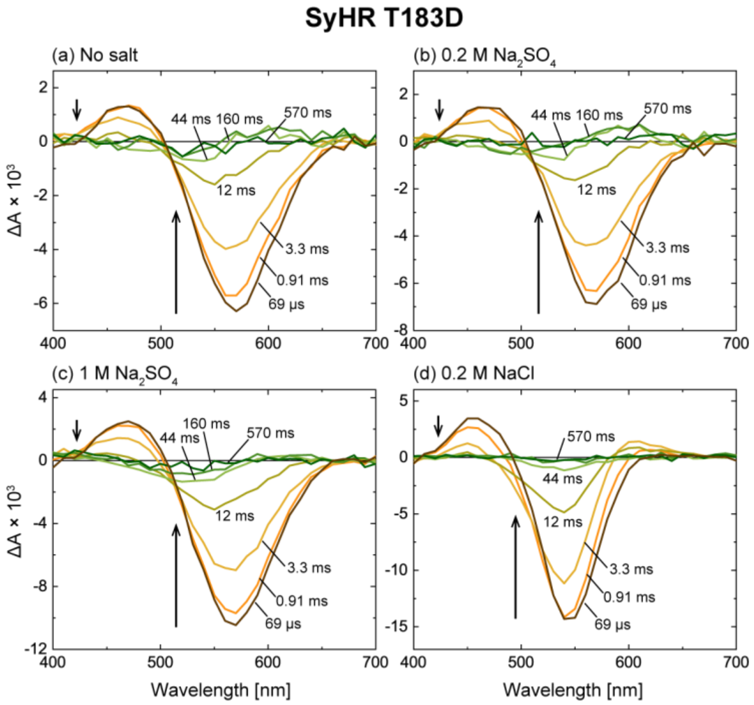

Figure S8. Light minus dark difference spectra of the T183D SyHR mutant. The sample preparations and the measuring conditions were the same as those of Fig. S7.

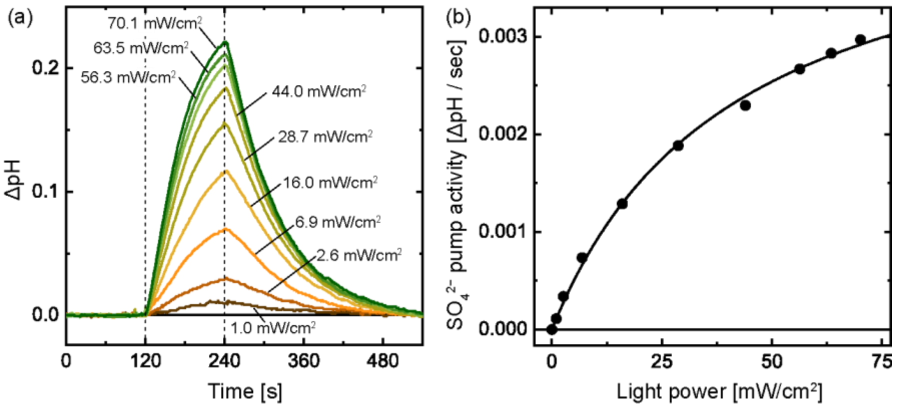

Figure S9. Illumination intensity dependence of SO_4^{2-} pumping activity of the E182T/F-helix MrHR. (a) Time courses of light-induced pH changes are plotted with the respective light intensities. (b) The initial slopes of pH changes are plotted against the light intensity.

2. Validity of the method for estimating the Cl^- pump amounts in *E. coli* cells

In this study, relative expressions of Cl^- pumps were estimated by the magnitudes of the flash-induced absorbance changes at 540 nm of the *E. coli* lysates (see Materials and Methods). 540 nm is a wavelength close to λ_{max} of MrHR and SyHR. Thus, the magnitudes of the negatively deflected signals at 540 nm reflect the expression amounts of the Cl^- pumps. However, this method requires the assumption that mutations do not significantly distort the photochemical properties of Cl^- pumps, including the quantum efficiencies of the photoreactions, the photoreactions themselves, and the extinction coefficients of dark states and intermediates. This assumption seems reasonable. This is because when we measured the Cl^- pump activities of the wild-types and mutant Cl^- pumps, we observed light-induced pH changes of similar magnitude (Fig. 2). To emphasize the validity of this method, we confirmed that there are no significant distortions in the photoreactions and the molar extinction coefficients in the dark states for representative mutants.

Figure S10 compares the flash-induced absorbance changes among the wild-type proteins and the representative mutants. For these measurements, we prepared the acrylamide gels containing *E. coli* membrane fragments expressing the respective proteins. Thus, Fig. S10 shows the results of a separate experiment from the estimation of protein expression amounts. In Fig. S10, time-courses of absorbance changes at three selected wavelengths are shown in the respective left panels. For calculations of the relative protein amounts, we picked up the maximum values of negatively deflected signals at 540 nm, whose time points are indicated by the vertical broken lines in the left panels. The respective right panels show the difference spectra at those time points. All the difference spectra have nearly the same shapes, indicating that the photoreactions are nearly identical, at least at those time points.

Figure S11 and Table S1 are the experimental results of obtaining the molar extinction coefficients (ε) in the dark state. Here, we prepared the purified proteins and measured the absorption spectra before and after the bleach reaction by hydroxylamine (HA). The black lines in Fig. S11 are the initial absorption spectra. The rhodopsin bands were bleached under illumination for 30-40 min in the presence of HA. After these treatments, the absorption bands of retinal oxime appeared in the absorption spectra (red lines) instead of the rhodopsin bands. The ε value of the retinal oxime is known to be 33,900 M^{-1} cm^{-1}. Thus, the ε values of the rhodopsins can be determined by comparing the absorbance values of rhodopsin bands with those of retinal oxime bands. The results are summarized in Table S1. Differences from wild-type protein values were within ±2.8% for MrHR mutants and within ±4.6% for SyHR mutants. These results also indicate that there are no significant distortions of photochemical properties due to mutations.

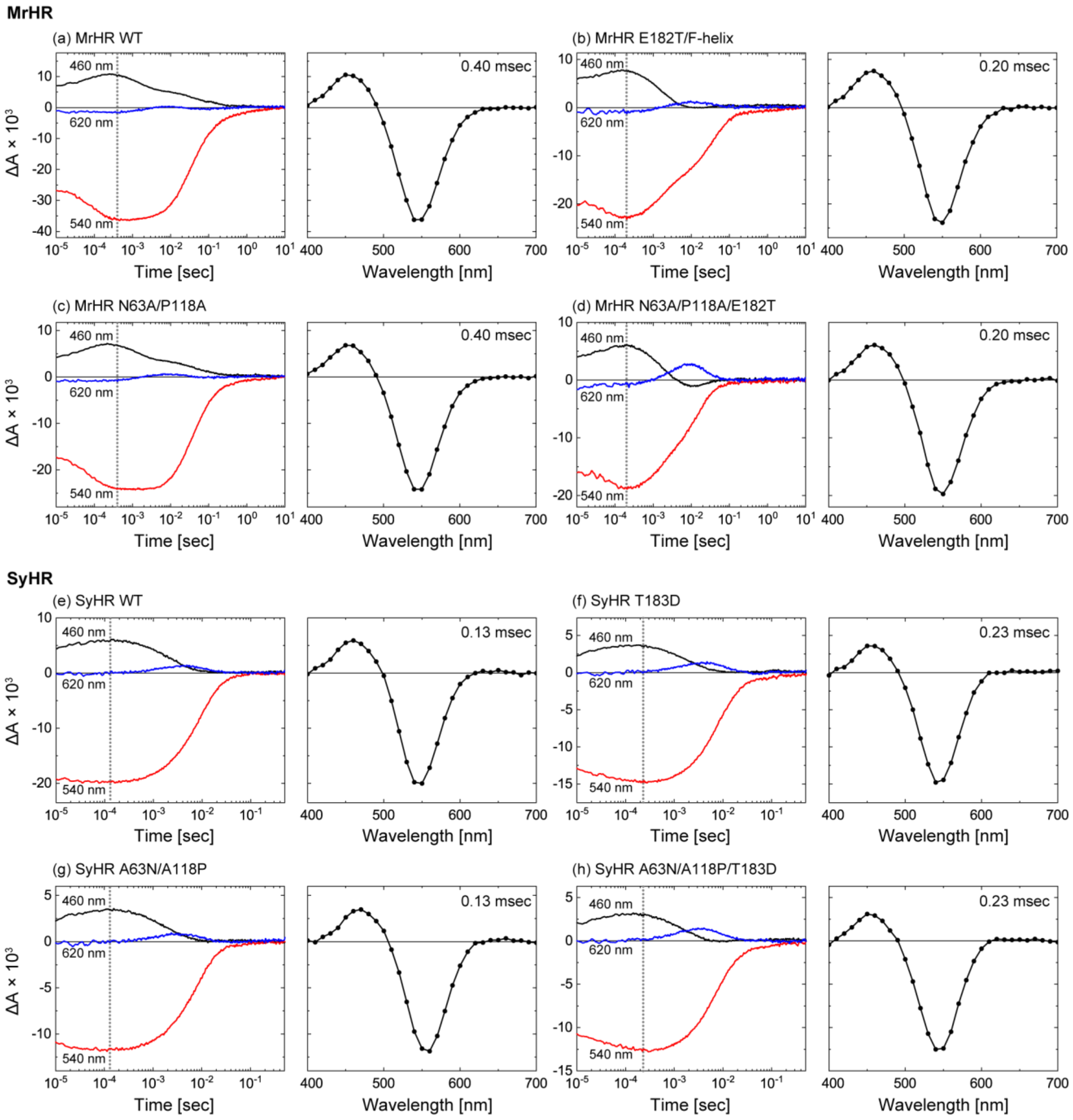

Figure S10. Comparisons of photoreactions among the wild-type proteins and the representative mutants. The left panels show the time-courses of flash-induced absorbance changes at three typical wavelengths, and the right panels show the difference spectra at the times points when the negative deflected signals at 540 nm became almost maximum values. These time points are shown in the right panels and are also indicated by the vertical broken lines in the left panels. The *E. coli* membrane fragments were used for the samples after encapsulation into 15% acrylamide gels. The buffer solution was 50 mM citric acid, pH 6.0, containing 0.2 M NaCl. For wild-type MrHR and the E182T/F-helix mutant, the data set shown in Fig. 6 and Supplementary Fig. S7 were also used here.

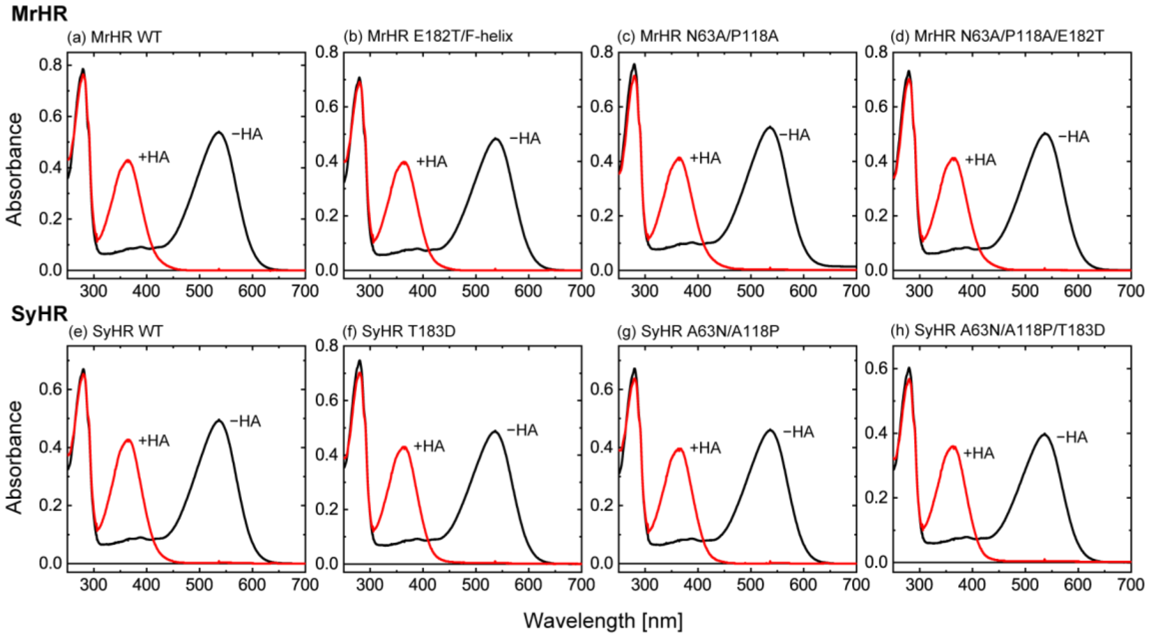

Figure S11. Absorption spectra of the wild-type proteins and the representative mutants before and after bleaching reaction with hydroxylamine (HA). The black lines labeled with "-HA" are the initial absorption spectra of the purified proteins, suspended in 50 mM citric acid, pH 6, containing 0.2 M NaCl and 0.05% DDM. These proteins were bleached for 30-40 min under illumination after adding a final concentration of 0.5 M hydroxylamine. The light source was a combination of a 650 W halogen lamp and two glass filters (IRA-25S, Y-46; Toshiba, Tokyo, Japan). After these treatments, the absorption bands of the retinal oxime appeared in the absorption spectra (red lines labeled with "+HA") instead of the rhodopsin bands. All experiments were performed at room temperature around 25 °C.

Table S1. Molar extinction coefficients (ε) at respective absorption maxima (λ_{max})

	λ_{max} [nm]	ε [M^{-1} cm^{-1}]
MrHR WT	536.5	42,400
MrHR E182T/F-helix	537.5	41,300
MrHR N63A/P118A	535.5	42,900
MrHR N63A/P118A/E182T	538.0	41,200
SyHR WT	537.5	39,100
SyHR T183D	536.5	38,500
SyHR A63N/A118P	537.0	39,600
SyHR A63N/A118P/T183D	536.0	37,300

The ε values were calculated from the spectra in Supplementary Fig. S11 according to the following equation:

$$\varepsilon = 33{,}900 \times \frac{A_{\text{-HA}}}{A_{\text{+HA}}}$$

where 33,900 is the ε value of the retinal oxime, $A_{\text{-HA}}$ and $A_{\text{+HA}}$ represent the absorbance peaks of rhodopsin and retinal oxime at around 536 nm and 360 nm, respectively.
